# Mental Disturbances in Turbinate Hypertrophies, or Nasal Stenosis

**Published:** 1898-07

**Authors:** John F. Woodward

**Affiliations:** Norfolk, Va.


### MENTAL DISTURBANCES IN TURBINATE HYPERTROPHIES, OR NASAL STENOSIS.

BY JOHN F. WOODWARD, M.D.,

NORFOLK, VA.

At first sight the title of this paper might convey to you the idea of a more profound study of the conditions accompanying nasal stenosis than the writer is prepared to discuss. Seven years ago, when I first began to study this subject, it was comparatively new; but to-day one is astonished at the literature on a subject thought to be new when he takes the time to investigate. Therefore I will not attempt to give a citation of the latest authorities, nor an historical sketch of its growth and influence upon the medical mind in the last few years. A decade of facts has placed it beyond the possibility of a hypothesis, and fixed it permanently in the chapter of scientific realities.

Guye, of Amsterdam, launched forth into medical nosology the term *aprosexia,* though I think the word *aphelia* (absence of mind, abstraction, revery and inattention) would have been equally as significant. Nevertheless, to Guye are we indebted for marking this condition as a distinct *mental aberration.*

Although I have never seen a case of maniacal fury nor ferocious insanity result from this state, yet I believe its further study will open up avenues of investigation that may solve to a great extent many of the quixotic, as well as seemingly malicious, deeds of our fellow-man.

For I know of no single ailment that will so soon and so radically change a patient's spirits and disposition from peace to unrest, from restlessness to irritability, and from irritability to suicidal and murderous tendencies. And, too, this condition leads many patients to magnify and brood over other troubles that might otherwise be forgot. This I have seen in three of my lady patients. It is well known that similar symptoms in the same person at different times mean different things, as there is an alarming varia-

*Read by title before the Southern Section of the Otological, Rhinological, and Laryngological Association, Atlanta, March 28.

bility in human beings as to their ability to place and appreciate physical abnormalities. Thus day by day it dawns upon us that there are endless chains of symptoms manfesting themselves, undercurrents as it were, whose meaning is lost in the agonies of real pain or smothered in the charity of ignorance and idiopathies.

The nose feels keenly the influences of the advances of civilization—change of modes of living, change of climate, and disturbance of the equilibrium of oxygen and moisture in the air by sweeping away our forests and shades and substituting arid fields and hot streets. Nasal disturbances are more common and their influence upon other portions of the system more manifest. Man is a complex being, and is not composed of any isolated special organs. Every part is in relation somehow and at some time with every other part, making the animate being an anatomical unit wherein the totality is but the centripetal and centrifugal influence of a motorial and sensorial automaton.

Understand the two nervous systems, and you have the key to the citadel; for, outside of the solar-plexus and the uterine reflexes, there is no field more fertile in reflexes than the nose and its accessories.

But I would defeat the object of this paper were I dwell too long upon reflex neuroses; I shall merely refer to them *en passant*, as I am thoroughly convinced that most of the mental manifestations in nasal hypertrophies are the result of toxic poisoning, either auto-genetic or hetero-genetic.

I have a long list of instructive cases, but will only give a few to help substantiate my remarks:

CASE 1.—Mr. P. A.; aged 27. Consulted me in 1891 about what he termed a "peculiar mental faculty." He had been a drinking man, but when I saw him he was very moderate in his habit. In the daytime he was sluggish, selfish, melancholic, morose, and at times very irritable and easy to anger. He brooded over imaginary troubles, and I have heard him speak of the relief death would afford. He had no insane hobby; and when night came he would bloom out into one of the most complete social successes I ever saw—bright and brilliant. I found a decided hypertrophy of both inferior turbinates, anterior and posterior. At times a great deal of watery discharge, and never a clear and open nose. This he had had for years, and looked upon it as natural, and had been using a little cocain in his nose every night, hence his change in disposition at that time. He was entirely relieved by treating and reducing the turbinates. There were no reflexes of importance, as he seldom had as much as a headache.

CASE 2.—Miss S. C. saw me in 1892 about her nose. She was not robust, but not a sickly person; aged 24, of healthy parents; she had never had a severe spell of sickness; looked stupid and inert, and her friend who came with her said she was "queer." I found enlargement of both inferior and middle turbinates on the left side, with pressure of the inferior against a cartilaginous spur. The spur was removed and the swollen turbinate reduced, with the restoration of the patient to a bright and cheerful young lady. She had neuralgias, migraine and ocular distress—all reflexes. But her toxic symptoms took the nature of melancholia and suicide, and at times she had hystero-cataleptic spells, though never very profound.

CASE 3.—Miss M. C.; aged 30; stenographer; family history good. For weeks she has been feeling dull and drowsy, crabbed and dissatisfied with everything, so much so that her friends became alarmed about her. Her employer said that her spelling and writing were so bad that he would have to give her up. She found in copying her dictation that words and sentences were incomplete. She had immense enlargement of the inferior turbinates, blocking up the nose almost completely at times; naso-pharyngitis with free mucous discharge. I gave no nerve sedatives, simply treated the nose with cautery and chromic acid, with a relief of all her discomfort in about six weeks.

CASE 4.—Miss B. A.; aged 20; stenographer; health good. She had gotten so bad that her physician suggested a complete rest from all work. She was excitable and irritable, and said that her employer was cruel to her, also that people laughed at her wherever she went; constantly expected something serious to happen to her; could not write an intelligible letter; had been treated for migraine and neuralgia for years. She had had adenoids removed many years ago. Turbinates enlarged and boggy; left middle turbinate pressed against the septum. The same treatment relieved her, but it took six weeks or more. No sedatives were used.

Mrs. M.; aged 35; mother of three children, with good general health. Beautiful and intelligent, but at her first visit to my office she was a wreck, mentally and physically—hysterical, suspicious, had secret warnings of impending dangers, such as loss of money and divorce; thought her husband was untrue, and longed for death; asked me to give her a slow poison so that she might kill herself. I found the same nasal trouble as in No. 4, except the turbinates were tense and redder. I treated her with chromic acid by *point burning* for two weeks, seeing her every third day, and then once a week for three weeks, at which time she was as well as ever.

CASE 6.—Mr. R. T.; aged 39; single; health good except slight dyspepsia and malaria. He used to drink very hard, but has been a total abstainer for five or six years. In 1896 he began to get nervous and fanciful; had exalted imagination with peculiar impressions as to bodily harm; the simplest physical ailment presented a fatal disease; afraid to go about by himself, afraid to use telephone, imagined that it would kill him; stopped reading newspapers because he had all the troubles that he read about; absolutely hopeless about himself, and no will-power. I had almost complete control over him by suggestion. His left nostril was blocked with a spur on the cartilaginous septum; hypertrophy of inferior and middle turbinates, and a granular pharyngitis with free secretions. Removing the spur and reducing the turbinates have almost entirely relieved him.

CASE 7.—Mrs. S. T. G.; aged 30; mother of four children; history good; attractive aud much beloved socially. Comes to see me twice a week for treatment. Right inferior and left middle turbinates enlarged. She says, "Really, doctor, I cannot think at all, and my mind gets confused when my nose stops up, and I lose my temper and am so irritable."

CASE 8.—Miss S. C.; aged 24; seamstress. Came to see me in regard to headache, dizziness, flighty spells, great depression, loss of memory, horrible dreams and general inertia. Middle turbinate of left side pressing hard against the septum; the inferior turbinate of same side is enlarged in its posterior aspect against a deflected septum. Treating the nose relieved her of all except the headache and the dizziness; these were cured with proper glasses.

CASES 9 AND 10.—A. R. and C. J., aged 7 and 5. Both victims of adenoids, and were called "dunce" at school; were cross and morose, stupid, and would not play like other children; with mouths open most of the time, they look like idiots. One operated upon in '96 and the other in '97; now they both look healthy and play and study like other boys.

You will notice that all of these cases had some form of neurosis with more or less mental manifestations—local or cephalic paresthesia (Dana), neuralgias, migraine, melancholia, aprosexia, listlessness, dread of some awful impending calamity, loss of will-power and fear of self, irritable and loss of confidence in fellow-man, emotional hysteria and suicidal tendencies; at times mentally bright and clear, but soon becoming morose, irritable and careless, a period when imaginary dangers might easily so impress the mind with imminent calamity that the welfare of others might be jeopardized.

I might cite many cases, but my time is limited. The cases referred to seem to suggest a predominance of mental manifestations over those of reflex neurosis. Thus I hope to stimulate the idea that these peculiar mental derangements are not so much the result of reflex neurosis as direct circulatory and metabolistic disturbances taking place in the brain from the absorption of hetero- or auto-genetic toxins. Constant or periodical exhibitions of functional mental derangements are pretty good evidence that there is some latent cell or fiber irritation, though the molecular change may be slight.

The majority of my cases were intumescent, hypertrophic or hyperplastic enlargements of the inferior and middle turbinates, and in patients otherwise healthy.

It is well known that the cavernous sinuses of the turbinates, undergoing either fibrous or mucoid changes, destroying or distending the sinuses, do not stop at the turbinates, but extend to the bone cells, affording a good field for absorption of toxic materials, or starting a neuro-vascular disturbance in neighboring tissue. High arterial tension in the brain will cause insomnia, and this high tension is generally compensated for by absorption of cerebro-spinal fluid. Cannot this pressure be kept up and the absorption prevented by metabolistic disturbances?

Cumston, in his excellent paper on auto-toxemia, refers to Mere, Bosc, Reges, Chevalier and others, who suggest that many mental diseases are due to auto-toxemia. Thus, instead of so many idiopathies, we must admit that there is absorbed into the blood certain products of internal chemical changes whose presence produce clinical signs only attributable to interference or excitation of certain functional organic processes, wherein the circulatory and central nervous system take a most active part.

There may be differences of opinion as to the nature and number of bacteria and micro-organisms in the nose, but there is a very decided belief that they are there, and especially in diseased noses. Our methods of nasal surgery go to prove that we fear to neglect a single chance to render the nose aseptic. Admitting the presence of germs in the nose and their power of generating toxins (ptomaines), along with Flatau's demonstration of the communication of the nasal lymph channels with the subarachnoid space, thus agreeing with Schwalbe, Key, Schreiber and others, seem to suggest that the osmotic and the endosmotic currents are potent factors in these cases. These encephalopathies seem to be toxic in nature. In diabetes, kidney diseases and typhoid fever, toxic absorptions produce mental disturbances.

Haubold, in the *N. Y. Medical Journal*, December, 1897, writing of self-intoxication, speaks of the different neurasthenias that arise therefrom, and has this to say: "Most frequent of all are perhaps the manifestations in the central nervous system, occurring as they do in many degrees, from the most trivial to the severest and most prolonged symptoms—cephalalgia, vertigo, syncope, insomnia, anxiety, stupor, coma, irritability, delirium, restricted spasms and general convulsions, epileptoid seizures, paralysis, and, not infrequently, hypochondria, melancholia, and mania." Pick, of Prague, believes in mental disorders and aberrations occurring as complications of nasal diseases, and reports cases. Daly reports 25 and Foster 3 cases neurasthenia cured by nasal treatment. Many cases of epilepsy have been reported, and some cures.

Adenoids are the only derangements of the nasopharynx that are apt to present mental manifestations, and their damage to early childhood cannot be estimated. Arrowsmith reports 700 cases of adenoids, many of whom manifested symptoms of headache, asthma, choreiform and epileptiform attacks, convulsions, and many diseases of speech. White, in Burnett's system for 1893, says: "There is no question about the improvement effected in the mental condition of many children by removing adenoids."

T. E. Hopkins says: "In mild cases the child looks stupid and is so. This stupidity has, according to A. Jacobi and Solis-Cohen, an anatomical basis in the relation between the lymphatic circulation through the brain and nasopharynx."

Spicer, of London, writing of the influence of nose and throat disorders upon children, says: "First, that certain nose and throat affections in children are among the special causes of derangements of sleep, temper, spirits, energy, intellectual powers, and other functions of the nervous system. Second, that long-standing derangement of these functions in the growing child implied a defect in the nutrition of tissue which would leave its mark on the nervous and mental faculties of the grown adult. Third, that the nasopharyngeal affections capable of causing the above derangements were chronic catarrh, with erectile and hypertrophied conditions of the mucous membrane."

Thus it seems to be adenoids and hypertrophic rhinitis that cause these mental manifestations. And I hope to elicit, by a further discussion, that a toxemia rather than a reflex neurosis acts as the excitant.

*35 Granby St.*

---

### Facial Erysipelas.

℞ Ac. carbolici........................................................

Tinct. iodi.............................................................

Spts. rectificat.....................................................āā ℥ i.

Ol. terebinth.......................................................... ℥ ii.

Glycerini.............................................................. ℥ iii.

M. Sig.: The lesions to be painted with this every two hours and covered with antiseptic gauze.—*La Presse Médicale.*

—*Med. Rev. of Reviews.*

